# Mechanisms of ribosome recycling in bacteria and mitochondria: a structural perspective

**DOI:** 10.1080/15476286.2022.2067712

**Published:** 2022-04-29

**Authors:** Savannah M. Seely, Matthieu G. Gagnon

**Affiliations:** a Department of Biochemistry and Molecular Biology, University of Texas Medical Branch, Galveston, TX 77555-1019, USA; b Department of Microbiology and Immunology, University of Texas Medical Branch, Galveston, TX 77555-1019, USA; c Sealy Center for Structural Biology and Molecular Biophysics, University of Texas Medical Branch, Galveston, TX 77555-1019, USA; d Institute for Human Infections and Immunity, University of Texas Medical Branch, Galveston, Texas 77555, USA

**Keywords:** Ribosome, protein synthesis, ribosome recycling, translation factors, elongation factor G, ribosome recycling factor, tRNA, HflX, GTPBP6

## Abstract

In all living cells, the ribosome translates the genetic information carried by messenger RNAs (mRNAs) into proteins. The process of ribosome recycling, a key step during protein synthesis that ensures ribosomal subunits remain available for new rounds of translation, has been largely overlooked. Despite being essential to the survival of the cell, several mechanistic aspects of ribosome recycling remain unclear. In eubacteria and mitochondria, recycling of the ribosome into subunits requires the concerted action of the ribosome recycling factor (RRF) and elongation factor G (EF-G). Recently, the conserved protein HflX was identified in bacteria as an alternative factor that recycles the ribosome under stress growth conditions. The homologue of HflX, the GTP-binding protein 6 (GTPBP6), has a dual role in mitochondrial translation by facilitating ribosome recycling and biogenesis. In this review, mechanisms of ribosome recycling in eubacteria and mitochondria are described based on structural studies of ribosome complexes.

## Introduction

In all organisms, the genetic information in messenger RNAs (mRNAs) is decoded and translated into proteins by a universally conserved macromolecular machine, the ribosome. The bacterial ribosome is composed of ~4,500 nucleotides and more than 50 ribosomal proteins, which assemble into a 70S ribosome made of two subunits, the small (30S) and the large (50S) subunits. The translation cycle is divided in four steps, initiation, elongation, termination, and recycling. Each step requires its own set of translation factors which interact with the ribosome in a sequential manner to control the accuracy and rate of protein synthesis.

The initiation complex begins with the 30S subunit, which binds the mRNA and selects the start codon positioned into the peptidyl (P) site. Assisted by initiation factors IF1, IF2, and IF3, the initiator fMet-tRNA_i_^Met^ binds the P site with high affinity and base pairs with the AUG codon on the mRNA [1]. Joining of the 50S subunit is catalyzed by IF2, a GTPase that regulates the maturation of the 70S initiation complex into an elongation-competent ribosome [2,3]. Following dissociation of initiation factors, the ribosome is now programmed with the initiator fMet-tRNA_i_^fMet^ in the P site and the first codon in the mRNA resides in the aminoacyl (A) site. The elongation cycle begins with the delivery of an aminoacyl-tRNA (aa-tRNA) by EF-Tu [4]. Decoding of the A-site codon stimulates hydrolysis of GTP by EF-Tu, which releases aa-tRNA into the A site [5–7]. After peptide bond formation, translocation of mRNA and tRNAs is catalyzed by elongation factor G (EF-G) and GTP [4,8–12]. Through a series of conformational changes in EF-G [13–15] and in the ribosome, including head swiveling of the 30S subunit [16–19] and ribosome ratcheting [8–10,20], tRNAs are translocated by one codon after each amino acid addition to the nascent polypeptide chain. Finally, the stop codon is recognized by release factors RF1 or RF2 [21–26]. Recognition of the stop codon triggers a conformational change in the release factor from its compact to extended conformation which allows its GGQ domain to dock into the peptidyl transferase center (PTC) near the nascent peptide chain attached to the peptidyl-tRNA in the P site, triggering hydrolysis and release of the complete protein [21–26]. Release factor 3 (RF3), found in a broad range of bacteria including *Escherichia coli*, facilitates the removal of RF1/RF2 from the ribosome [27–29]. The termination complex is then recycled into individual ribosomal subunits by EF-G, GTP, and the ribosome recycling factor (RRF) [30–33].

Over the last two decades, structures of key ribosome complexes have been elucidated with the use of X-ray crystallography and cryo-electron microscopy (cryo-EM), providing important insights into the mechanisms of protein synthesis. While the steps of initiation, elongation, and termination have received considerable attention, structures of ribosome complexes undergoing recycling remain relatively scarce. The lack of high-resolution structures of functional ribosome complexes before and after subunit splitting has impeded our understanding of this essential step of protein synthesis. In this review, we describe the current state of understanding of ribosome recycling that emanated from the structures of bacterial and mitochondrial ribosomes complexed with recycling factors.

### RRF is a structural mimic of tRNA

**Figure 1.**
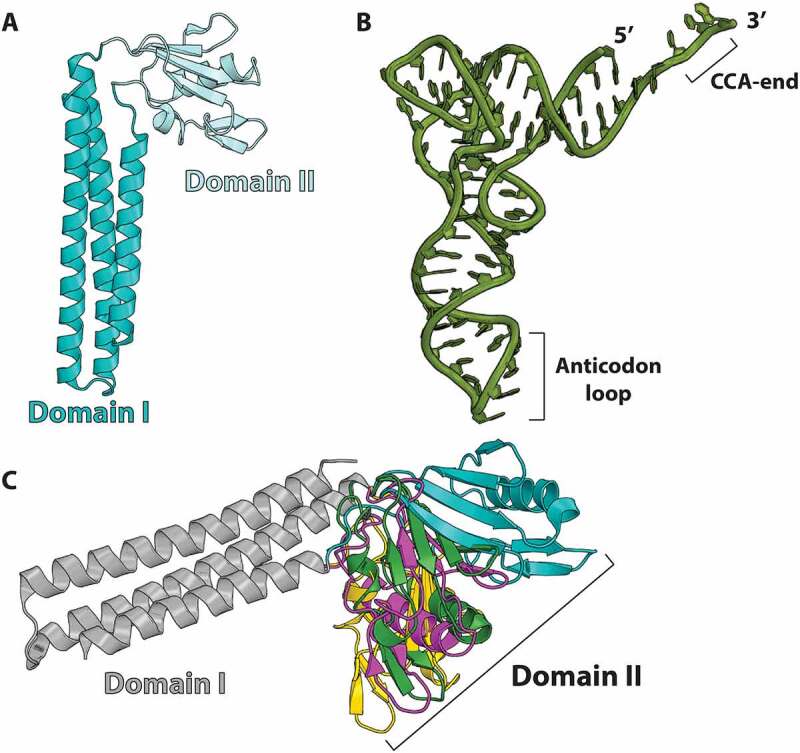
RRF is a tRNA mimic with a flexibly disposed domain II. (A) Ribbon diagram of the *E. coli* RRF crystal structure (PDB 1EK8) [37]. Domains I and II are distinctly colored and connected by flexible linkers. (B) L-shaped structure of tRNA. (C) Crystal structures of RRF aligned by domain I show that domain II rotates about the axis of domain I (PDBs: 1EK8, teal; 1DD5, gold; 1EH1, green; 1GE9, magenta) [37–40].

In bacteria, EF-G is typically a dual function protein that in addition to catalyzing tRNA and mRNA translocation, also promotes ribosome recycling. Ribosome recycling by EF-G requires RRF, the inactivation of which was shown to be lethal in *E. coli* [34,35]. It was initially proposed that RRF binds to the A site of the ribosome, similarly to release factors RF1 and RF2 [36]. Crystal and solution NMR structures of RRF revealed that its fold mimics that of the tRNA L-shape [37–40]. RRF consists of two domains, a long triple α-helix coil-coil bundle domain (domain I), and a smaller α/β domain (domain II) (Fig. 1A, B). Alignment of the RRF structures through the triple-helix bundle domain I reveals that both domains in RRF are linked through flexible linkers, allowing domain II to freely rotate around the long axis of domain I (Fig. 1C) [37,41]. Domain swapping experiments in RRF demonstrated that domain II plays a crucial role in recycling the ribosome presumably through its interaction with EF-G [42]. Hydroxyl radical probing of RRF bound to the *E. coli* 70S ribosome suggested that despite the fact that the structure of RRF mimics that of tRNA, the orientation of RRF in the ribosome differs significantly from the binding position of tRNA [41]. The model proposed that the long triple helix bundle domain I of RRF binds across the A and P sites on the large subunit (LSU), thereby overlapping with the positions of the acceptor arms of the A- and P-site tRNAs in the 70S ribosome.

The first cryo-EM reconstruction of the 70S ribosome bound to RRF provided, albeit at a low-resolution, a glimpse of the binding site of RRF at the interface of the subunits of the ribosome [43]. The structure essentially confirmed the previous binding position of RRF inferred from hydroxyl radical probing protection experiments [41], placing domain II further toward the small subunit (SSU), and revealed conformational changes in the inter-subunit bridge B2a that is formed between helix H69 of 23S rRNA and the top of helix h44 of 16S rRNA. This cryo-EM structure provided a rationale for the role of RRF in facilitating dissociation of the ribosomal subunits. Helix 69 plays a functional role in many steps of protein synthesis, including subunit association and tRNA binding. Large ribosomal subunits lacking helix H69 are unable to associate with the small subunits to form functional ribosomes, and the assembled 70S ribosome can be recycled in the absence of RRF, demonstrating the importance of bridge B2a for the stability of the 70S ribosome [44]. In agreement with the disruption of bridge B2a during ribosome recycling, a crystal structure of the *Deinococcus radiodurans* 50S subunit complexed with domain I of RRF showed that the tip of H69 moves by 20 Å toward h44 of the SSU [45]. However, the physiological relevance of the 50S-RRF crystal structure raised doubts because RRF itself preferably binds to the 70S ribosome over the 50S subunit [46,47]. Furthermore, RRF bound to the 50S subunit is not released by EF-G, the latter being required for efficient ribosome recycling [48].

**Figure 2.**
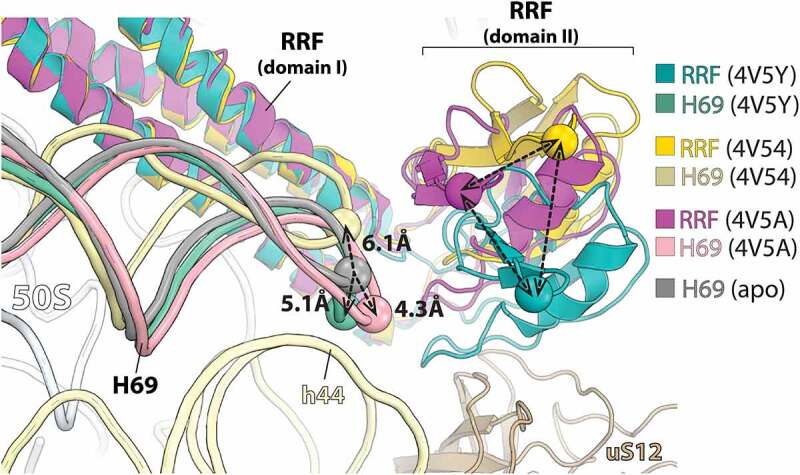
Domain II of RRF is flexibly disposed on the 70S ribosome. Crystal structures of RRF on the 70S ribosome aligned by 23S rRNA. In the absence of EF-G, domain II of RRF occupies different positions relative to the 23S rRNA helix H69 (PDBs: 4V5Y, *E. coli* 70S-paromomycin-RRF; 4V54, *E. coli* 70S-RRF; 4V5A, *T. thermophilus* 70S-RRF) [32,33].

The crystal structure of the *Thermus thermophilus* 70S ribosome bound to RRF showed that under the experimental conditions used, RRF does not induce H69 movement [32]. On the contrary, the crystal structures of the *E. coli* 70S bound to either *T. thermophilus* or *E. coli* RRF reported that RRF induces H69 movement away from the SSU h44 [31,33] (Fig. 2). The apparent discrepancy observed in the movement of H69 among the ribosome-RRF complex structures may be attributed to the absence of EF-G in these experiments. EF-G is required for RRF-mediated ribosome recycling and its influence on the conformation of RRF and the ribosome must account for its function.

**Figure 3.**
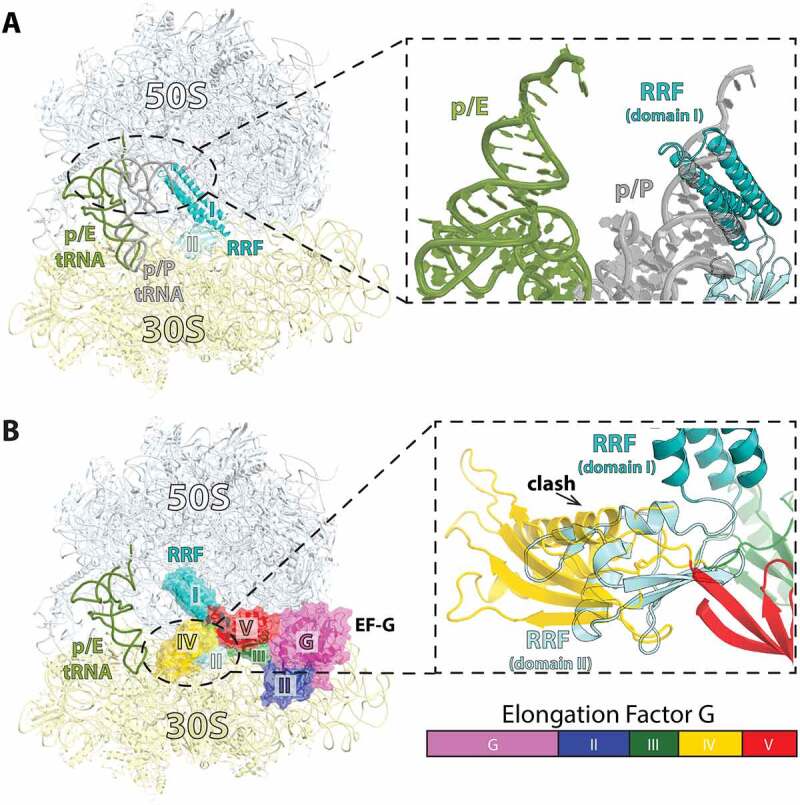
RRF in the post-termination complex (PoTC) is not compatible with tRNA in the p/P state of binding and EF-G on the 70S ribosome. (A) RRF (teal/light blue) bound to the 70S ribosome with p/E-tRNA (olive) (PDB 4V9D [56]). The p/P-tRNA (gray) is shown. Inset: Domain I of RRF clashes with tRNA bound in the p/P state. (B) Structure of EF-G-GDPCP in the extended state bound to the 70S ribosome (PDB 4V5F [4]). EF-G is colored by domain according to the bar chart. Inset: Domain IV of EF-G in the extended conformation is not compatible with RRF without further rotation of RRF domain II about the long-axis of domain I.

The ribosome is known to fluctuate between the ratcheted and non-ratcheted conformations. This refers to the rotation of the small subunit relative to the large subunit in the 70S ribosome. The ratcheting motion of the ribosome is thermally driven in that the ribosome can spontaneously sample both rotated and non-rotated conformations [49] and occurs in the absence of factor [50,51]. However, these fluctuations do not lead to productive translocation in the absence of EF-G. EF-G bound to GTP induces the rotated conformation of the ribosome [20], which is required for mRNA and tRNA translocation. However, the state of the ribosome to which RRF binds has remained unclear as cryo-EM and single molecule Förster resonance energy transfer (smFRET) experiments demonstrated that the association of RRF with a post-termination 70S ribosome containing a deacylated tRNA in the P site induces the ribosome to adopt the rotated state [52–54]. In agreement with RRF binding to the 70S ribosome following peptide release, RRF has low affinity for the non-rotated ribosome containing peptidyl-tRNA in the P site [55]. The crystal structure of RRF bound to a fully rotated *E. coli* 70S ribosome reported essentially the same interactions between domain I of RRF and the ribosome as with the non-rotated ribosome, while domain II interacts with ribosomal protein uS12 and is more constrained in the rotated ribosome [56]. In this structure, the acceptor stem of the deacyl-tRNA has moved to the E site of the LSU and the tRNA is bound in the p/E hybrid position due to SSU rotation, which effectively avoids a steric clash between the triple helix bundle domain I of RRF and the acceptor stem of deacyl-tRNA (Fig. 3A). The orientation of domain II on the rotated ribosome suggests that RRF must undergo large rearrangements to co-exist on the ribosome together with EF-G (Fig. 3B). The presumed rotation of domain II in RRF induced by EF-G would lead to conformational changes in regions of the 50S subunit that are involved in inter-subunit bridging (e.g. bridge B2a).

The presence of mRNA and deacyl-tRNA on the post-termination ribosome was shown to increase the rate of subunit splitting by the concerted action of RRF and EF-G [57]. Yet, the structure with RRF bound to the rotated ribosome and deacyl-tRNA in the p/E hybrid state fails to explain how deacyl-tRNA facilitates subunit splitting. For instance, the same p/E-tRNA hybrid state is observed during EF-G-mediated tRNA translocation, which does not lead to ribosome subunit dissociation. Until recently, little remained known of the interactions that form between RRF and EF-G on the pre-recycling 70S ribosome, and the role of deacyl-tRNA in subunit splitting (see below).

### The concerted action of EF-G and RRF recycles the ribosome

EF-G and RRF act together to split the post-termination 70S ribosome into its individual subunits. It was proposed that IF3 also acted as a ribosome splitting factor [46]. However, further experiments confirmed that IF3 is not required for ribosome splitting, but rather associates with free 30S subunits and serves the role of an anti-association factor, keeping SSU from re-associating with free LSU [58,59].

RRF bound to the ribosome without EF-G is observed to occupy two locations at the interface of the subunits, one that is same as previously determined, and a new position exclusively on the 50S subunit overlapping with that of tRNA in the P site [60]. Although the low-resolution cryo-EM structure suggested that RRF may ‘spontaneously’ move across the inter-subunit space disrupting contacts between the ribosomal subunits, the action of EF-G during recycling remained unclear. The lack of structures of pre-recycling 70S ribosome complexed with both RRF and EF-G is due to the rapid splitting of the ribosome (~5 sec^−1^ *in vivo*) by these two factors [61]. Structural studies rely on the formation of stable complexes with lifetimes that are compatible with the experimental approach used to visualize it. Crystallization of the ribosome is a time consuming process and complexes that are not stable enough represent a challenge for structure determination using X-ray crystallography [62–65]. The technique of cryo-EM represents an advantage over crystallography in that it bypasses the crystallization step, and can be used to capture structural intermediates and less stable complexes. The recent ‘resolution revolution’ in cryo-EM has opened a realm of new possibilities enabling visualization of large protein machineries at near-atomic resolution, which is essential to the understanding of how nanomachines function. The use of cryo-EM has been fueled by developments of transmission electron microscopes optics, software for data analysis, and sensors that combine fast readouts with the ability to directly detect electrons [66,67]. Ribosome complexes are assembled, applied to a holey-mesh carbon grid, flash-frozen in a thin film of vitreous ice, and single particles are visualized by electron microscopy (EM). Time-resolved cryo-EM is being developed and shown to be valuable to capture short-lived intermediates of ribosome complexes undergoing fast transitions, allowing reconstructions of functionally relevant transient structures [3,7,12,21,30,68].

The first structure of a post-termination ribosome in complex with both RRF and EF-G used heterogeneous factors and ribosome. Cryo-EM was used to reconstruct structures of a complex containing the 70S ribosome and EF-G from *E. coli*, and RRF from *T. thermophilus* [69]. The structures revealed new interactions between domain II of RRF and the ribosome in the absence of EF-G, forming contacts with helices H43 and H44 in the uL11-stalk of the 23S rRNA, part of the GTP-activating center (GAC). With EF-G bound, domain II of RRF rotates towards the 30S subunit, locating in the vicinity of inter-subunit bridge B2a as observed in other structures. The interpretation of these results was, however, obscured by the fact that this heterogeneous combination of factors is inactive in ribosome recycling [70,71].

Time-resolved cryo-EM was used to trap the ribosome incubated with RRF, EF-G and IF3 during subunit splitting [30]. The ribosome was rapidly mixed with RRF, EF-G and IF3, and the grids frozen. At the 140 ms reaction time point, four types of complexes were observed. The first class shows rotated 70S bound to RRF and with tRNA in the p/E state. In this complex, domain II of RRF is in contact with protein uS12 as observed in the crystal structure of rotated *E. coli* 70S ribosome bound to RRF [56], contrary to the heterogeneous complex in which domain II orients toward the 50S subunit [69]. The second class contains RRF bound to the non-rotated ribosome without tRNA. Compared to the rotated ribosome, domain II interacts with the stalk base (GAC) of the 50S subunit, reminiscent to the interaction previously described for the recycling complex formed with heterogeneous factors [69]. However, it was suggested that this class is not an authentic intermediate in the recycling process due to the lack of tRNA in the map. The third class has both EF-G and RRF bound to a rotated ribosome with a tRNA in the p/E state. The low resolution of these structures (~7.5–16 Å) makes it difficult to unambiguously determine the location of domain II of RRF because its density appears fused with that of EF-G. Yet, the angle between domains I and II decreased by ~60° as domain II rotates toward helix h44 of SSU and loses interaction with protein uS12. Domain IV of EF-G, the A-site binding domain during tRNA translocation, is seen to contact domain II of RRF, while domain III of EF-G is unresolved in this map. As expected from the ribosome splitting reaction, individual 30S and 50S subunits were also observed. The SSU is either bound to tRNA or IF3, and the LSU remains associated with EF-G and RRF. On the LSU, domain I of RRF occupies the same position as that seen on the 70S-RRF and 70S-RRF-EF-G complexes. Domain II, however, is rotated even further toward helix H69 of 23S rRNA when compared to the 70S-RRF-EF-G complex. Taken together, these results corroborated previous observations indicating that EF-G assists the movement of domain II of RRF towards bridge B2a and jointly acts with RRF to split the post-termination complex into individual subunits.

**Figure 4.**
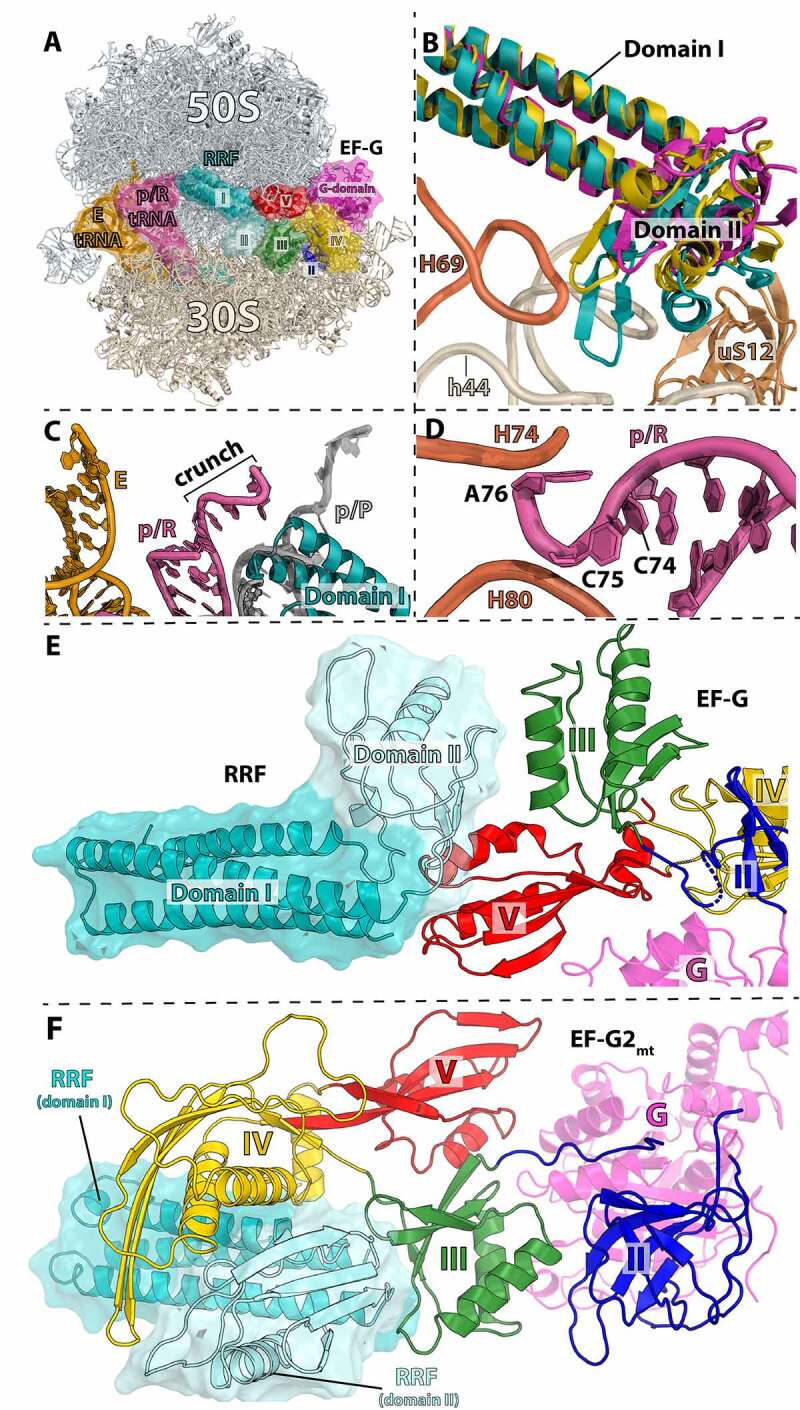
Pre-recycling complex with p/R- and E-site tRNAs. (A) Overview of pre-recycling complex (PDB 6UCQ [62]) with E-site tRNA (orange), p/R-tRNA (pink), RRF (teal and light blue), and EF-G in the compact state (colored by domain). (B) RRF domain II positioned in a ‘ready-to-attack’ state. Domain II (teal) locates in the niche created by H69 (orange), h44 (cerium), and uS12 (brown). RRF from crystal structures in the absence of EF-G superimposed through domain I of RRF (PDBs 4V5A, gold; 4V55, magenta) [32,33]. (C) Close-up view of the tRNA interaction with RRF domain I wherein the p/R-tRNA CCA-end is crunched and displaced by ~22 Å toward the E site and exhibits shape complementarity with RRF. The classical p/P-tRNA is not be compatible with RRF on the 70S ribosome. (D) The CCA-end of the p/R-tRNA is squeezed between 23S rRNA helices H74 and H80 (orange). (E) Interactions between compact EF-G and RRF. Domain II of RRF interacts favorably with EF-G domains III and V. (F) Interactions between RRF_mt_ and EF-G2_mt_ in the post-recycling complex (PDB 7L20 [115]) wherein EF-G2_mt_ has undergone rearrangements of domains III, IV and V. Domain IV of EF-G2_mt_ forms favorable interactions with the surface of RRF_mt_ domain II, which has rotated to avoid a steric collision with EF-G.

The structures described above provided important insights into the mechanism of ribosome splitting by RRF and EF-G. Yet, the role of tRNA in facilitating this process remained unclear. In all of the recycling complexes that carry a tRNA the same p/E hybrid conformation was observed. The hybrid p/E state of tRNA binding occurs all the time during EF-G-mediated tRNA translocation and even just when the ribosome spontaneously takes the rotated state. Thus, how can the rate of subunit splitting by RRF and EF-G be 15-fold faster with tRNA bound to the post-termination ribosome [57]? The crystal structure of a pre-recycling complex bound to RRF, EF-G, and two tRNAs provided a plausible explanation to this conundrum [62]. In this study EF-G bound to GDP stabilizes the ribosome in a non-rotated state complexed with RRF and tRNAs in the P and E sites. As expected, domain I of RRF occupies the same position as previously seen on the non-rotated *E. coli* and *T. thermophilus* 70S ribosome [32,33]. The position of the acceptor stem of P-site tRNA is not compatible with the simultaneous binding of RRF domain I on the 50S subunit (Fig. 3A). Consequently, the deacyl-tRNA is tilted toward the E site and the CCA-end is located halfway between the P and E sites on the 50S subunit (Fig. 4A, C). The CCA-end of the p/R-tRNA is blocked by a constriction formed by helices H74 and H80 of 23S rRNA (Fig. 4D). The 3’-terminal nucleotides of tRNA are crunched together, suggesting that the tension that builds up in tRNA may facilitate subunit splitting. This data supports the notion that, despite the absence of tRNA translocation during ribosome recycling [72,73], splitting of the ribosome proceeds rapidly in the presence of deacyl-P-site tRNA [57,73]. Correspondingly, RRF interacts weakly with translating ribosomes carrying peptidyl-tRNA in the P site[55], and EF-G and RRF do not dissociate such ribosomes [73].

The presence of EF-G in this crystal structure complex causes domain II of RRF to rotate toward helix H69 of 23S rRNA as seen by time-resolved cryo-EM [30]. Compared to the crystal structures of RRF-70S complexes [32,33], EF-G pushes domain II deeper into the space formed between H69 and ribosomal protein uS12, suggesting a ‘ready-to-attack’ state of RRF on the central bridge B2a (Fig. 4B). In this pre-recycling complex, EF-G adopts a compact conformation identical to the one previously reported on a pre-translocation 70S ribosome [15]. In this conformation, domain IV of EF-G is directed away from RRF, and domains III and V of EF-G form a cleft into which RRF domain II docks [62]. In the previous cryo-EM structures of the 70S-RRF-EF-G [30] and 50S-RRF-EF-G [52,74], domain IV of EF-G lies on top of RRF. One major difference is that in the 70S-RRF-EF-G structure [30], the ribosome is rotated. Thus, EF-G would presumably undergo a large conformational rearrangement from the compact to the extended form as the ribosome transitions to the rotated state (Fig. 4E, F).

The compact form of EF-G is likely transient, being trapped on the ribosome because of the intermolecular contacts in the crystal that lock the ribosome in the non-rotated state. The rapid transition of the ribosome to the rotated state in solution makes compact EF-G difficult to capture by cryo-EM. Single molecule FRET experiments suggested the existence of a compact EF-G on the ribosome [13], and a low-resolution cryo-EM reported large domain movements in EF-G on the ribosome [14]. Despite this, recent time-resolved cryo-EM studies of EF-G bound to the 70S ribosome during tRNA translocation did not observe the compact form of EF-G [11,12,75], further suggesting that it is not a ribosome-EF-G state that is highly populated. EF-G in its extended conformation interacts with RRF on the post-termination 70S complex; however, the low-resolution of the available cryo-EM studies limits the interpretation of the specific contacts between EF-G and RRF, and with the ribosome. Structures of pre-recycling ribosome complexes determined at higher resolution are required for a better understanding of ribosome recycling.

### Select bacteria harbor multiple copies of EF-G

The genome of several bacteria contains more than one copy of the gene encoding for EF-G [76,77]. However, there are limited studies into the function and mechanism of these additional homologues. EF-G2 in *T. thermophilus* exhibits ribosome dependent GTPase activity and low levels of elongation activity in poly(U)-dependent protein synthesis while its possible role in recycling remains unclear [78]. The function of EF-G2 in *Mycobacterium smegmatis* remains ambiguous due to the lack of GTPase activity which renders it unable to participate in elongation or recycling [79]. *Borrelia burgdorferi* EF-G1 and EF-G2 have been determined to have specific singular activity rather than being bi-functional, wherein EF-G1 functions exclusively in elongation and EF-G2 functions exclusively with RRF in recycling, similarly to the suggested specific activity for the EF-G1A and EF-G1B homologues identified in *Pseudomonas aeruginosa* [80,81]. Currently, it remains difficult to derive conclusions regarding the specialization of EF-Gs in bacteria due to the absence of structural information. High-resolution structures of bacterial ribosomes complexed with specialized EF-Gs will be required to understand how specific forms of EF-G function exclusively during elongation while other forms work concomitantly with RRF during ribosome recycling.

### HflX is an alternative ribosome recycling factor

**Figure 5.**
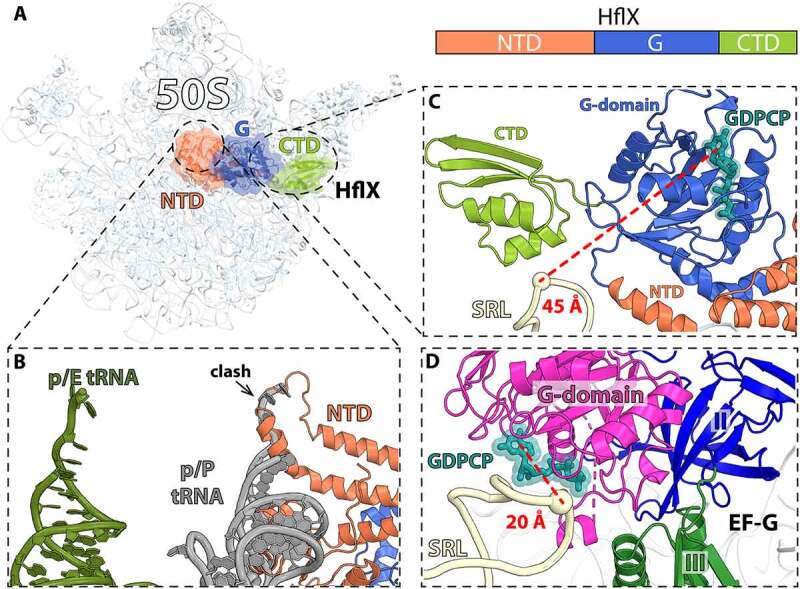
Cryo-EM structure of *E. coli* 50S subunit bound to HflX. (A) Overview of *E. coli* HflX bound to the 50S subunit (PDB 5ADY [91]). (B) Close-up view of the HflX N-terminal domain with superimposed p/P- and p/E-tRNAs showing that the p/P-tRNA is not compatible with HflX. (C) The GDPCP nucleotide in the G-domain of HflX locates ~45 Å away from the 23S rRNA sarcin-ricin loop (SRL). (D) In EF-G-GDPCP bound to the 70S ribosome, the GDPCP nucleotide in the G-domain is closer (~20 Å) to the SRL (PDB 4V9H [8]).

**Figure 6.**
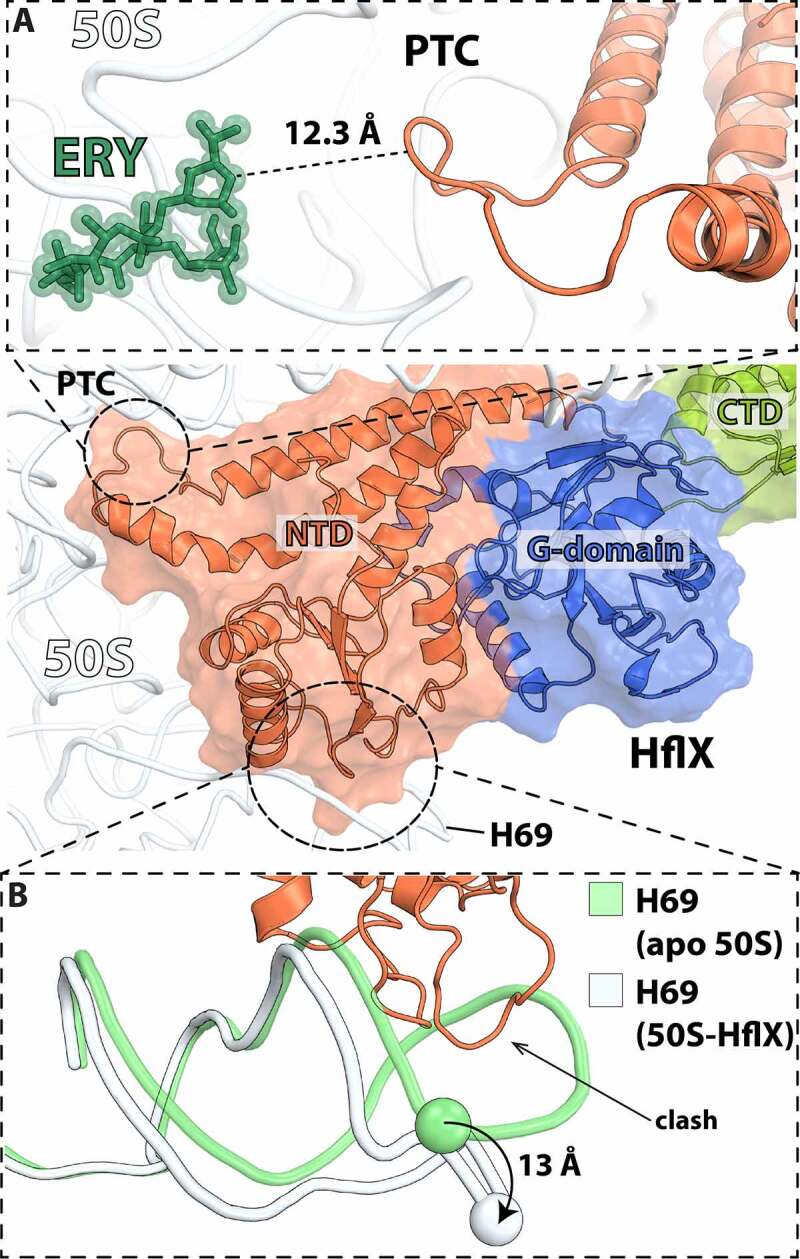
Interactions of HflX with the 50S ribosomal subunit. (A) Close-up view of the PTC loop within the HflX N-terminal domain (orange) (PDB 5ADY [91]) with the antibiotic erythromycin (ERY) (green) bound in the nascent peptide exit tunnel (NPET) (PDB 6ND6 [130]). The nearest distance between HflX and ERY is more than 12 Å. (B) The N-terminal domain of HflX (orange) displaces 23S rRNA helix H69 by ~13 Å (white) relative to the apo form (green) of the 50S subunit.

In bacteria, HflX is one of the 11 conserved GTPases and shares high sequence homology with the ODN protein family (Obg, DRG1 and Nog1) involved in ribosome assembly [82]. Like the ODN family proteins, HflX binds to LSU in a nucleotide dependent manner, including GTP, GDP, ATP, and ADP [83–85]. However, only the GTP-hydrolysis activity of HflX is stimulated upon ribosome binding [83]. Yet, under conditions that favor regeneration of nucleotide diphosphate into triphosphate forms, it was shown that ATP hydrolysis by HflX is stimulated by the 70S ribosome and free 50S subunits [86]. Despite being universally conserved, HflX is dispensable in *E. coli* under normal growth conditions [87]. The *hflX* gene is part of a complex superoperon, *amiB-mutL-miaA-hfq-hflX-hflK-hflC*, characterized by genes that are co-transcribed from a series of alternating Eσ^70^ and Eσ^32^ heat shock promoters [88,89]. The relative amount of *hflX* transcript increases ~5-fold in cells undergoing heat shock [88]. HflX rescues stalled ribosomes during early elongation steps [90] and rapidly restores translational capacity to the cell during heat shock response. HflX in *E. coli* has been described to rescue stalled ribosomes by splitting the 70S ribosome into subunits, effectively recycling them at a rate that is around 5-fold slower than that with EF-G, RRF, and IF3 [91]. HflX exhibits a three-domain structure; the GTPase domain, the C-terminal domain (CTD), and the N-terminal domain (NTD) that is made up of two sub-domains [91]. While chemical crosslinking experiments have previously suggested that HflX binds near the ribosomal E site, structure determination by cryo-EM showed that HflX binds along the subunit interface covering the A site and overlapping with the P site (Fig. 5A) [91,92]. HflX binding in this position would clash with a peptidyl-tRNA in the P site and accounts for the observed lower splitting efficiency when a peptidyl-tRNA is present in the 70S (Fig. 5B) [91]. However, a deacyl-tRNA in the p/E-hybrid position would be accommodated and therefore, it is likely that HflX has preferential binding for a rotated ribosome similar to RRF and EF-G [91]. When in complex with the 50S subunit, and unlike other GTPases, the G-domain of HflX is positioned in such a way that it does not contact the sarcin-ricin loop (SRL) of the 50S subunit, suggesting that GTPase activation in HflX occurs by a completely different mechanism compared with other translational GTPases (Fig. 5C, D) [83,86,91]. Structure alignment of the 50S:HflX:GDPNP cryo-EM reconstruction with the 50S subunit of the 70S ribosome shows that HflX causes rearrangements of H69 in LSU such that it would collide with the SSU h44 (Fig. 6B). This suggests that similar to canonical recycling by EF-G and RRF, disruption of the contact between H69 and h44 is used by HflX to dissociate the 70S ribosome. However, structures of HflX in complex with the 70S ribosome will be necessary to elucidate this further.

Recently, HflX homologues in *Mycobacterium abscessus* and *M. smegmatis* have been associated with resistance to lincosamide and macrolide antibiotics [93,94]. Expression of *M. abscessus* and *M. smegmatis* is under the control of the WhiB7 transcriptional activator which upregulates the expression of the *erm* genes in the presence of sub-inhibitory concentrations of antibiotics [95,96]. HflX-ribosome dependent splitting was also observed in these species; however, HflX was unable to prevent H^3^-erythromycin (ERY) from binding to the ribosome or remove it from the LSU [94]. The NTD of HflX extends toward the PTC, with the tip of the NTD located ~12.3 Å from erythromycin bound deeper into the nascent peptide exit tunnel (NPET), which may explain why HflX fails to dislodge ERY from the LSU (Fig. 6A). It was suggested that HflX alone is not sufficient to mediate antibiotic resistance and that a second factor may be required to remove the bound antibiotic from the 50S subunit before it can undergo a new round of translation [94].

*Listeria monocytogenes* carries two *hflX* genes, and one was named *hflXr* because its expression is associated with resistance to lincomycin and ERY. The *hflXr* gene is under the control of the rli80 leader sequence and when exposed to lincomycin the transcription of *hflXr* significantly increases due to transcription attenuation control of rli80 associated genes [97]. Deletion of the *hflXr* gene leads to increase sensitivity to lincomycin and ERY while its over-expression increases resistance. HflXr is proposed to recycle antibiotic-stalled 70S ribosomes because they accumulate in *hflXr* knockout cells exposed to sub-lethal concentrations of ERY [97]. High-resolution structures of HflX/HflXr in complex with the 70S ribosome and 50S subunit are needed to elucidate the mechanism by which HflX recycles stalled ribosomes. Additionally, clarity is needed as to how HflX and HflXr mediate resistance through ribosome recycling and what additional factors may be involved in the prevention of antibiotic binding or antibiotic removal from the 50S subunit.

### Recycling of the mitoribosome

Eukaryotic cells contain two distinct and separate translation systems with unique machinery, one localized to the cytoplasm and one localized to the mitochondrial matrix, except in the case of plants which contain an additional translational system localized to chloroplasts. Mammalian mitoribosomes are distinct from both cytosolic mammalian ribosomes and bacterial ribosomes but are more reminiscent of the latter as mitochondria are thought to have originated from an endosymbiotic event between primitive eukaryotic cells and α-protobacterium [98,99]. Mammalian mitochondria carry their own genomic DNA coding for 13 essential subunits of the oxidative phosphorylation system critical for maintaining mitochondrial function in mammalian cells, as well as 22 tRNA and two ribosomal RNA genes [100]. Several human disorders have been attributed to mitochondrial translation deficiencies [101]. 55S mitoribosomes are composed of large (39S) and small (28S) subunits containing 16S and 12S rRNA, respectively, and over 80 ribosomal proteins. In contrast with their bacterial counterparts, mammalian mitoribosomes are made up of approximately 70% protein and the mass attributed to rRNA is significantly reduced [102,103]. Correspondingly, structure comparison of the *Saccharomyces cerevisiae* (yeast) mitoribosome with that of the human and porcine mitoribosomes shows that while the yeast mitoribosome appears to be on an evolutionary trajectory that has not experienced rRNA contraction, the situation is opposite for mammalian mitoribosomes for which the evolutionary path is the contraction of rRNA and the increase of ribosomal protein mass [102,104–109]. Despite these variations, the four steps of translation are conserved in mitochondria such that once synthesis of the nascent peptide is terminated and the peptide released, the mitoribosome must be recycled for use in a new round of initiation. Similar to the bacterial counterpart, the mitoribosome is recycled by the concerted action of mitochondria-specific RRF and EF-G. It has been shown that deletion of mitochondrial RRF (RRF_mt_) is lethal to mammalian cells and causes mitoribosome aggregation, loss of oxidative phosphorylation complex, and a rise in mitochondrial superoxide production [110].

**Figure 7.**
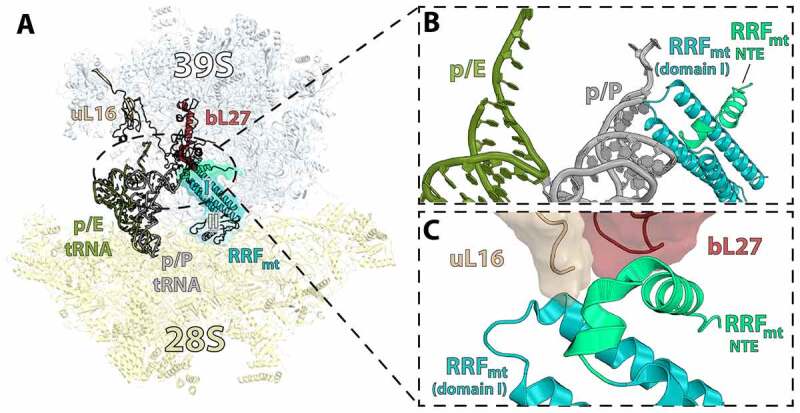
Binding of RRF_mt_ on the post-termination mitoribosome is stabilized by large subunit interactions and is not compatible with tRNA in the p/P state. (A) RRF_mt_ (teal/light blue/light green) bound to the 55S ribosome (PDB 6NU2) with p/E-tRNA (olive) and p/P-tRNA (gray) shown (PDB 7NSJ and 7NSI respectively) [112,116]. (B) Domain I of RRF_mt_ is not compatible with tRNA bound in the p/P state. (C) The N-terminal extension (NTE) of RRF_mt_ exhibits stabilizing interactions with large subunit mitoribosomal proteins uL16 and bL27 (tan and brown respectively).

The human mitochondrial RRF_mt_ has approximately 25–30% sequence identity to that of bacterial RRF with one major distinction being that RRF_mt_ harbors an N-terminal extension (NTE) that is 80 amino acids in length [111]. Co-immunoprecipitation experiments with RRF_mt_ alone showed an association with mitoribosomal proteins from both the 39S and 28S subunits, suggesting that RRF_mt_ binds to the 55S mitoribosome [110], which was later confirmed by cryo-EM. The structure of RRF_mt_ bound to a model post-termination mitoribosome at 3.9-Å-resolution shows that the body of the 28S subunit is rotated by ~8.5^°^ relative to the 39S subunit [112]. This rotation is comparable to the ratcheted state of the bacterial ribosome wherein the 30S subunit rotates counterclockwise 5–10^°^ with respect to the 50S subunit [20]. Additionally, the head of the 28S subunit also rotates in an orthogonal direction towards the E site, similar to swiveling of the head domain observed in the bacterial ribosome [17,51,56,112]. RRF_mt_ binding to the rotated mitoribosome is suggested to ‘prime’ the mitoribosome for splitting by stabilizing a state in which 7 of the 15 inter-subunit bridges are broken or destabilized, including 3 mito-specific bridges [103,106,107]. RRF_mt_ binds along the inter-subunit space and exhibits similar size and domain composition to that of its bacterial counterpart with the NTE extending from the tip of the triple-helix bundle domain I (Fig. 7). The tip of RRF_mt_ domain I is positioned close to the PTC such that a peptidyl-tRNA in the P site would clash with RRF_mt_. While in most bacterial ribosome structures domain II of RRF is positioned at a right angle relative to domain I, the same angle in RRF_mt_ is more open and domain II is located in close proximity to mitochondrial small subunit protein uS12m [112]. The last 21 residues of the RRF_mt_ NTE are resolved in the RRF_mt_:55S structure and positioned perpendicular to the tip of RRF_mt_ domain I [112]. A cluster of rRNA helices as well as ribosomal proteins uL16m and bL27m are in close proximity to the RRF_mt_ NTE. The N-terminal end of bL27m is known to be flexible and is not well resolved in most bacterial ribosome structures. In the RRF_mt_ bound structure, the NTE interacts with bL27m and shows that the N-terminal of bL27m occludes the P site and would interfere with a peptidyl-tRNA in the P site. This interaction between RRF_mt_-NTE and bL27m led to the suggestion that it may stabilize binding of RRF_mt_ to the 55S mitoribosome (Fig. 7) [112]. The NTE also interacts with the 16S rRNA of the 39S subunit near the conserved A-site loop that binds the CCA-end of the incoming aminoacyl-tRNA [112]. The RRF_mt_ NTE may block the A and P sites ensuring that tRNAs and translation factors do not bind to the mitoribosome during recycling. We note that the N-terminal extension of RRF_mt_ has the propensity to adopt different conformations and to form alternative interactions in the mitoribosome [113].

Following binding of RRF_mt_, EF-G bound to GTP is required to dissociate the mitoribosome into subunits and release the tRNA and mRNA. Mammalian mitochondria harbor two homologues of EF-G: EF-G1_mt_ and EF-G2_mt_. Both EF-G1_mt_ and EF-G2_mt_ have significant ribosome-dependent GTPase activity that is comparable to bacterial EF-G [114]. However, while bacterial EF-G is typically a bi-functional translation factor that participates in both elongation and recycling, it was suggested that each mitochondrial EF-G homologue has a singular function, similarly to what has been reported in *B. burgdorferi* [81]. Single round translocation experiments in a reconstituted *in vitro* mitochondrial translation system showed that only EF-G1_mt_ had robust translocation activity [114]. When the recycling activity of EF-G1_mt_ and EF-G2_mt_ was investigated in a polysome breakdown assay, EF-G2_mt_, and not EF-G1_mt_, caused accumulation of monosomes and individual subunits suggesting that EF-G2_mt_ is the GTPase that acts together with RRF_mt_ in mitochondria to recycle the mitoribosome [114].

**Figure 8.**
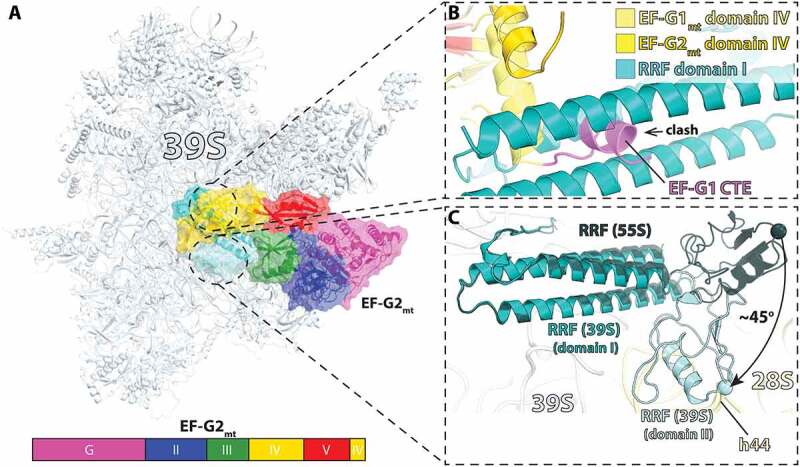
Cryo-EM structure of mitochondrial RRF_mt_ and EF-G2_mt_ bound to the 39S mitoribosomal subunit. (A) Overview of RRF_mt_ and EF-G2_mt_ bound to the 39S mitoribosomal subunit post-recycling (related to Fig. 4F) (PDB 7L20 [115]). (B) Close-up view of RRF_mt_ domain I interactions with EF-G2_mt_ domain IV (gold). The structure shows that the C-terminal extension (CTE) of EF-G1_mt_ (PDB 6VLZ, purple [117]) would collide with domain I of RRF, preventing EF-G1_mt_ from participating in the recycling step. (C) Superposition of RRF_mt_ from the 55S class I structure (dark turquoise, PDB: 7L08) [115] aligned by domain I on the class three complex with RRF_mt_ (teal) and EF-G2_mt_ (omitted for clarity) on the 39S, wherein RRF_mt_ domain II is rotated approximately 45° toward the SSU and exhibits a collision with the superimposed h44.

Ribosome recycling by EF-G2_mt_ has been investigated structurally by cryo-EM, wherein a model post-termination complex composed of a 55S mitoribosome was incubated with puromycin followed by incubation with RRF_mt_ and EF-G2_mt_. Puromycin is an aminonucleotide antibiotic causing premature chain termination, thus ensuring the P-site tRNA is deacylated. Three major classes were observed upon single-particle analysis: i) the 55S mitoribosome with RRF_mt_ bound, ii) the 55S mitoribosome with RRF_mt_ and EF-G2_mt_ bound, and iii) the 39S large subunit with RRF_mt_ and EF-G2_mt_ bound [115]. The class one structure determined here matches the previous 55S:RRF_mt_ cryo-EM structure from the same group described above [112]. However, additional density corresponding to the RRF_mt_ NTE was resolved and observed to be located in a pocket between domains I and II of RRF_mt_ (Fig. 7). This section of the NTE is also in close proximity to h44 from the small subunit, large subunit helices H69 and H71, and ribosomal protein uS12m. The NTE in this position was proposed to be critical to the stabilization of RRF_mt_ domain II by compensating for the shortened H69 in mitochondria compared to that of bacterial H69. The second class of mitoribosomes were observed to have clear density for RRF_mt_ and EF-G2_mt_ but the density for the 28S subunit was weak and unclear as to what the predominant conformation may be. This is likely due to the small number of particles that populated the second class of ribosomes containing both factors as the recycling reaction happens on a rapid time scale. When comparing the position of RRF_mt_ between the three classes, domain I remained in the same position while domain II was observed to rotate 45° toward the 28S subunit in class three versus its position in class one (Fig. 8A, C) [115]. A similar rotation (61°) was reported for domain II of RRF_mt_ bound to the porcine mitoribosome [116]. This rotation is mediated by the binding of EF-G2_mt_, and without such conformational change domain II would collide with domains III, IV, and V of EF-G2_mt_. The conformation of EF-G2_mt_ on the 39S with RRF_mt_ creates a pocket for the interaction of RRF_mt_’s hinge region and the loops of domain III in EF-G2_mt_. It was suggested that these interactions are essential to facilitate rotation of RRF_mt_ domain II and subsequent splitting of the 55S mitoribosome. EF-G2_mt_ domain IV is observed to be pressed against RRF_mt_ domain II and α-helix 3 of domain I, and these interactions were suggested to stabilize the rotated conformation of RRF_mt_ domain II.

While the general binding position and domain arrangements of EF-G1_mt_ and EF-G2_mt_ on the mitoribosome are similar, there are structural differences between the two isoforms that provide possible explanations for the specialized nature of their functions. Interestingly, EF-G2_mt_ was described to have a specific role in destabilization of the inter-subunit bridge B2a via a steric clash between loop 1 of domain IV and the SSU h44. In contrast, the domain IV loop 1 region of EF-G1_mt_ is oriented toward the decoding center away from h44 and therefore would not exhibit this clash [117,118]. However, differences in orientation of some regions are not the only substantial differences between EF-G1_mt_ and EF-G2_mt_. EF-G1_mt_ is smaller (83 kDa) than EF-G2_mt_ (87 kDa). EF-G2_mt_ has 36% sequence identity to EF-G1_mt_ and 30% to bacterial EF-G. There is significant divergence in the C-terminal ends in domain IV between the two mitochondrial isoforms, with EF-G1_mt_ containing a C-terminal extension that is not present in EF-G2_mt_ which is not compatible with domain I of RRF_mt_ on the ribosome (Fig. 8B) [115,116]. This may account for EF-G1_mt_’s lack of recycling ability. Differences in EF-G1_mt_ and EF-G2_mt_ bound to the ribosome also have shed light on EF-G2_mt_’s translocation deficiency. EF-G2_mt_ lacks one of two glycine residues in the loop 1 region of domain IV that are universally conserved in bacteria and are present in EF-G1_mt_ [115]. The glycine residues facilitate the tight turn of loop 1 required for interactions with the minor groove of the mRNA:tRNA duplex, critical for destabilization of the mRNA:tRNA duplex and facilitating translocation by one codon length. The second glycine residue is replaced by an aspartic acid in EF-G2_mt_ making it unfavorable for interacting with the mRNA:tRNA duplex and preventing its participation in elongation. Furthermore, the electrostatic potential of the molecular surfaces of EF-G2_mt_ and RRF_mt_ appears to facilitate their interaction, while EF-G1_mt_ would be electrostatically incompatible with RRF_mt_ causing the two moieties to repulse each other [116].

Yeast and plant mitochondria also contain their own localized translation systems that undergo the four conserved steps in translation, but the recycling step specifically has not been studied as extensively as above in mammalian mitochondria. The factors involved in yeast mitochondrial ribosome recycling have been identified and their role in mitochondrial DNA maintenance and stability have been investigated but their mechanism in ribosome splitting has not been the subject of structural or biochemical studies [119–121]. The study of yeast mitochondrial translation has been hindered by the lack of an *in vitro* translation system and the association of the yeast mitochondrial ribosome to the inner membrane [122].

### GTPBP6 is an alternative ribosome recycling factor in mitochondria

**Figure 9.**
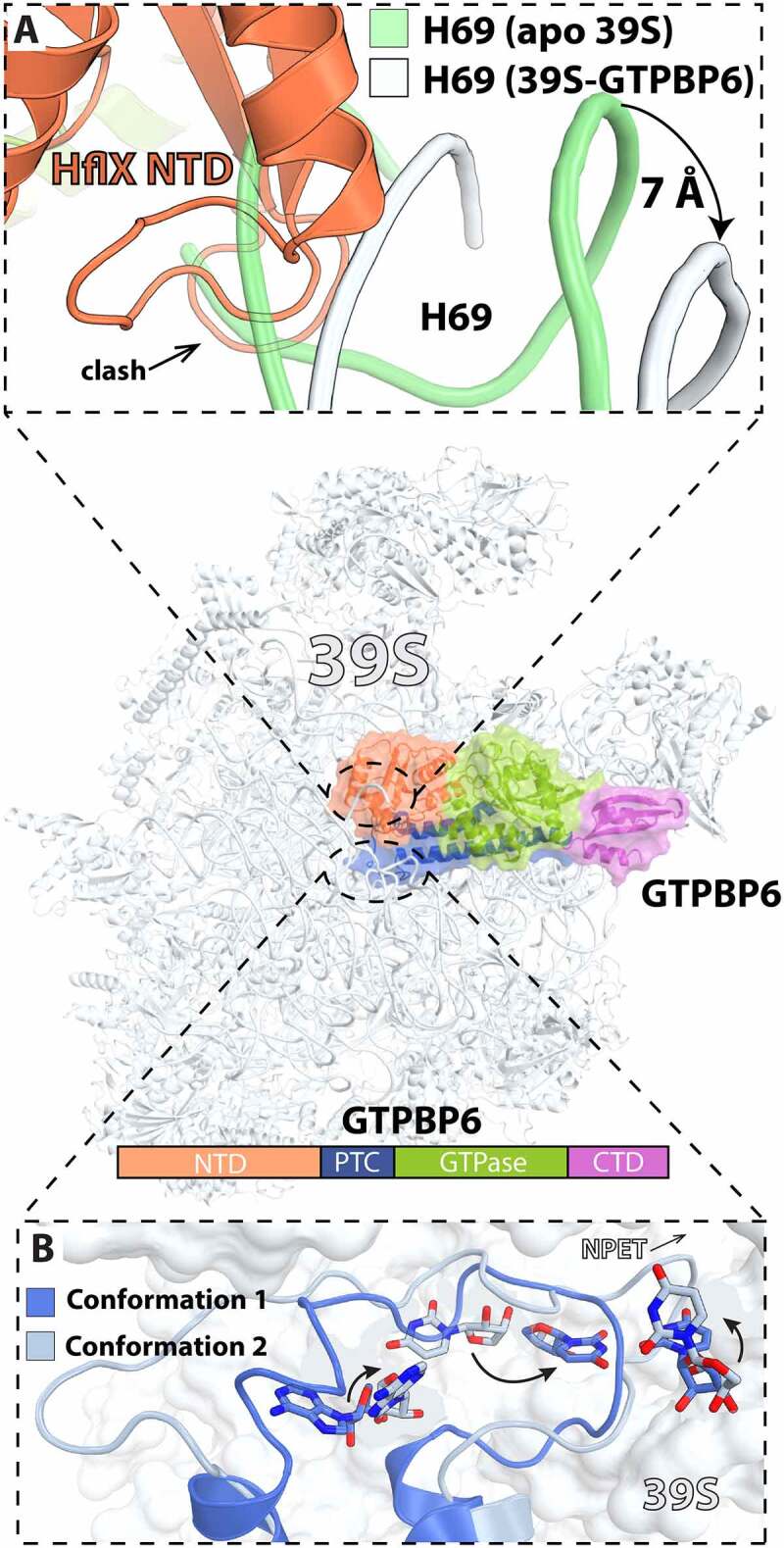
Cryo-EM structure of GTPBP6 bound to the 39S mitoribosome subunit. (A) Close-up view of the interactions between the GTPBP6 N-terminal domain (PDB 7OF4, orange [129]) and H69. Helix H69 in the apo 39S subunit is green (PDB 6NU3 [112]), showing that it is not compatible with the binding of GTPBP6, shifting by ~7 Å (PDB 7OF4, white [129]). (B) Two conformations of the PTC region upon GTPBP6 binding to the 39S mitoribosome subunit. Superimposition of the 39S subunit with PTC conformation 1 (PDB 7OF4, dark blue [129]) with that of the 39S subunit with PTC conformation 2 (PDB 7OF6, light blue [129]) reveals rearrangements of PTC residues A3089 (*E. coli* A2602), U3072 (*E. coli* U2585), and U2993 (*E. coli* U2506).

In eukaryotic cells, several highly conserved classes of GTPases localize to the inner membrane of mitochondria and serve a variety of functions related to quality control of 55S mitoribosome assembly [123–127]. One such mito-specific GTPase, GTP-binding protein 6 (GTPBP6), serves two functions: 39S subunit maturation during assembly and 55S ribosome recycling [128,129]. GTPBP6 is homologous to the bacterial HflX sharing approximately 30% sequence identity and a similar domain arrangement. Deletion of the *GTPBP6* gene leads to accumulation of mtLSU at a late stage of maturation resulting in mitochondrial translation defects. Elevated levels of GTPBP6 lead to the accumulation of mtSSU and mtLSU, suggesting GTPBP6 is involved in dissociation of 55S mitoribosomes. This was confirmed by sucrose density gradient ultracentrifugation experiments that showed dissociation of the 55S ribosome in the presence of GTPBP6, and by stopped-flow kinetics where the presence of GTPBP6 and GTP resulted in a significant reduction in light scattering [128]. GTPBP6 also contains an ATP-dependent RNA helicase domain that may be involved in GTPBP6ʹs mitoribosome biogenesis function but in the presence of ATP, GTPBP6 is unable to recycle the mitochondrial ribosome. As in *E. coli*, GTPBP6 ribosome splitting activity is significantly decreased with ribosomes containing a peptidyl-tRNA in the P site, suggesting that the human mitochondrial HflX homologue does not recycle actively translating mitoribosomes [128]. In a post-recycling state, GTPBP6 binds to the 39S subunit in a similar manner to that of bacterial HflX on the 50S subunit [129]. Similar to *E. coli* HflX, GTPBP6 contains a loop that extends toward the PTC, called the PTC-binding loop, which has been implicated in the maturation of the mitoribosome PTC during 39S biogenesis and mitoribosome assembly. The PTC-binding loop is observed in two distinct conformations, one of which is only observed in the context of recycling while the other is observed in recycling and biogenesis experiments. In conformation 2, the PTC-binding loop is extended into the PTC by rearrangements in α5, α7, and α8 (Fig. 9A, B). In conformation 2, highly conserved rRNA residues in the PTC are observed to take on a conformation that is seen during elongation and peptide release, but it is currently unclear whether GTPBP6 recognizes the PTC in this conformation or induces this change. In the 39S:GTPBP6 complex, similar interactions between GTPBP6 and H69 are similar to what is seen in the 50S:HflX complex, however the tip of H69 is not modeled in the GTPBP6:39S complex and therefore a detailed comparison of its movement is not possible at this time. The new position of H69 is thought to clash with h44 on the 28S subunit and suggests a similar mechanism for mitoribosome disassembly by GTPBP6 (Fig. 9A). Unlike its bacterial counterpart, GTPBP6 has not been implicated in rescue or antibiotic resistance. Possible GTPBP6 function under stress conditions may be a necessary avenue of investigation to fully understand its role in mitoribosome recycling.

### Concluding remarks

Structures of the ribosome complexed with canonical and alternative splitting factors have contributed to the molecular understanding of ribosome recycling. One common theme emerges: bacterial and mitochondrial ribosome recycling factors destabilize the central inter-subunit bridge formed between h44 in the small subunit and helix H69 in the large subunit. While conformational changes in RRF domain II that displace helix H69 are induced by EF-G, the N-terminal domain of HflX performs this function. Nevertheless, the significance of displacing H69 can only be speculated at this time in the absence of high-resolution structures of pre-recycling ribosomes complexed with recycling factors. Similarly, the molecular basis by which HflX-mediated ribosome recycling is associated with antibiotic resistance in *L. monocytogenes* [97] and *M. abscessus* [93,94] requires further analysis.
